# Application of few-shot learning and transfer learning based on YOLOv6 in the recognition of bacteria in sputum M-ROSE

**DOI:** 10.3389/fcimb.2026.1791986

**Published:** 2026-04-22

**Authors:** Yan Huang, Long Cao, Hongchen Xue, Meng Zhang, Yuanjun Liu, Xiaoping Huang, An Liu

**Affiliations:** 1 Department of Infectious Diseases, The First Affiliated Hospital of Soochow University, Suzhou, Jiangsu, China; 2 School of Computer Science and Technology, Soochow University, Suzhou, Jiangsu, China

**Keywords:** bacteria classification, deep learning, few-shot learning, M-ROSE, YOLOv6

## Abstract

**Background:**

Rapid pathogen identification is essential for guiding timely and appropriate antimicrobial therapy in severe pulmonary infections. Microbiological rapid on-site evaluation (M-ROSE) can provide preliminary etiological information at the bedside, but its interpretation is labor intensive and highly dependent on experienced clinicians or microbiologists. This study aimed to develop and evaluate a deep learning framework for bacterial identification in sputum M-ROSE smears under limited annotated data conditions.

**Methods:**

A total of 161 Gram-stained sputum M-ROSE images containing Acinetobacter baumannii, Klebsiella pneumoniae, and Pseudomonas aeruginosa were collected using a digital scanner. Thirty annotated images were used to construct Dataset N, which was divided into a training set and a validation set at a ratio of 8:2. A deep learning model based on the YOLOv6 architecture was developed using transfer learning and few-shot learning. In addition to conventional data augmentation, two customized strategies—rotation cutting and image fusion—were introduced to enhance the detection of extremely small bacterial targets. The remaining 131 images were used as an independent testing set. Model performance was evaluated using recall, precision, F1 score, mean average precision (mAP), accuracy, and diagnostic time.

**Results:**

The best-performing model achieved 89.31% recall, 84.23% precision, an F1 score of 0.8670, and an mAP of 0.817 on the validation set. On the independent test set, the model achieved 93.89% accuracy. In addition, the model required substantially less time for image interpretation than the participating clinicians.

**Conclusion:**

The proposed YOLOv6-based framework showed good performance for bacterial identification in sputum M-ROSE smears under limited annotated data conditions. These findings support the feasibility of applying data-efficient deep learning strategies to real-world clinical microbiological images and suggest potential utility in rapid microbiological diagnosis.

## 1 Introduction

Antimicrobial resistance (AMR) has become a major global public health challenge. In 2019, bacterial AMR was associated with an estimated 4.95 million deaths worldwide, including 1.27 million deaths directly attributable to resistant infections (Murray et al., 2022). The increasing prevalence of multidrug-resistant organisms further underscores the importance of rapid and accurate pathogen identification for guiding targeted antimicrobial therapy and improving clinical outcomes (Heames et al., 2020; Murray et al., 2022).

Microbiological rapid on-site evaluation (M-ROSE) is a smear-based diagnostic approach that has gained increasing attention in recent years. Its major advantage lies in its rapid turnaround time, as it can be performed at the bedside and combines immediate morphological assessment with microbiological pathogen identification. For patients with severe pulmonary infections, especially those in critical care settings, timely pathogen recognition is essential for early therapeutic decision-making. Previous studies have shown that sputum M-ROSE has good concordance with culture results and can provide preliminary etiological information substantially faster than conventional culture-based methods or next-generation sequencing (NGS) (Huang et al., 2021; Su et al., 2021; Xu et al., 2022). Therefore, sputum M-ROSE has substantial clinical value for the rapid etiological diagnosis of severe respiratory infections. In addition to informing antibiotic selection, dynamic monitoring of bacterial classification and burden in M-ROSE smears may help assess infection severity and provide visual evidence of treatment response.

Despite these clinical advantages, interpretation of sputum M-ROSE smears remains highly dependent on experienced clinicians or microbiologists. In routine practice, a large number of high-magnification microscopic fields must be examined, making the process labor intensive and time consuming. Moreover, inter-observer variability may affect diagnostic consistency. Because different bacterial species exhibit relatively distinct morphological characteristics under microscopy, M-ROSE provides a suitable basis for automated image-based recognition.

In recent years, deep learning (DL) has shown strong potential in medical image analysis and has increasingly been applied to microbiological imaging tasks. Previous studies have demonstrated that DL models can be used for automated bacterial detection, classification, and interpretation of microscopic images, thereby reducing manual workload and improving diagnostic consistency (Smith et al., 2018; Kang et al., 2019; Wang et al., 2021). However, the application of DL to clinical M-ROSE images still faces several important challenges. First, expert-annotated clinical image datasets remain limited. A recently published clinical bacterial dataset for M-ROSE further highlighted the scarcity of real-world clinically annotated images and benchmark datasets and algorithms in this field (Wang et al., 2024). Second, sputum M-ROSE images represent a typical small-data scenario in medical imaging, for which transfer learning and data augmentation have been recognized as effective strategies (Piffer et al., 2024). Third, bacteria in sputum smears are extremely small targets embedded in complex microscopic backgrounds, which substantially increases the difficulty of automated detection. Recent studies further suggest that DL can support multi-task bacterial image analysis and may extract morphology-related phenotypic information associated with antimicrobial resistance directly from light microscopy images (Chin et al., 2024; Ikebe et al., 2024).

To address limited annotated data, few-shot learning (FSL) and transfer learning (TL) have increasingly been introduced into medical image analysis (Pan and Yang, 2009; Finn et al., 2017; Snell et al., 2017; Raghu et al., 2019). Transfer learning enables models to leverage prior knowledge learned from large-scale source datasets for related target tasks, thereby reducing dependence on large annotated medical datasets (Pan and Yang, 2009; Raghu et al., 2019). Few-shot learning further aims to improve model generalization when only a limited number of labeled samples are available (Finn et al., 2017; Snell et al., 2017). These strategies are particularly relevant in clinical microbiology, where data acquisition and expert annotation are often costly and difficult. Nevertheless, robust deep learning frameworks specifically tailored to real clinical sputum M-ROSE images remain limited, particularly in scenarios involving extremely small bacterial targets and constrained annotation resources.

In this study, we developed a YOLOv6-based deep learning framework for bacterial identification in sputum M-ROSE smears. Building on transfer learning and few-shot learning, we combined conventional data augmentation with two customized strategies—namely, rotation cutting and image fusion—to enhance detection of extremely small bacterial targets in real clinical smear images. Using images containing Acinetobacter baumannii, Klebsiella pneumoniae, and Pseudomonas aeruginosa, we evaluated the diagnostic performance of the proposed framework and explored its potential as an AI-assisted tool for rapid microbiological diagnosis in clinical practice.

### 1.1 Data acquisition

#### 1.1.1 Acquisition of sputum M-ROSE smear data

The collection of sputum M-ROSE specimens, smear preparation, staining, and microscopy followed standardized laboratory protocols (National Health and Family Planning Commission of the People's Republic of China, 2017) (see **Supplementary Material** for details).

##### 1.1.1.1 Microscopy

**Figure 1:**
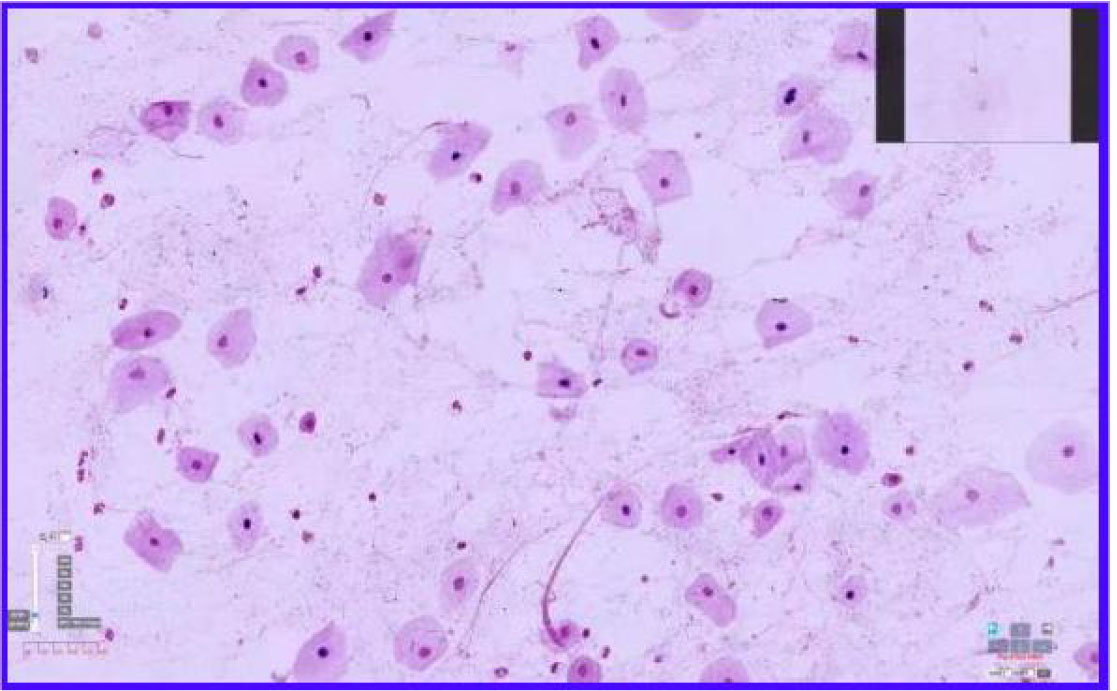
(Left) Non-qualifying sputum smear, showing a significant increase in squamous epithelial cells (×1500 magnification).

**Figure 2:**
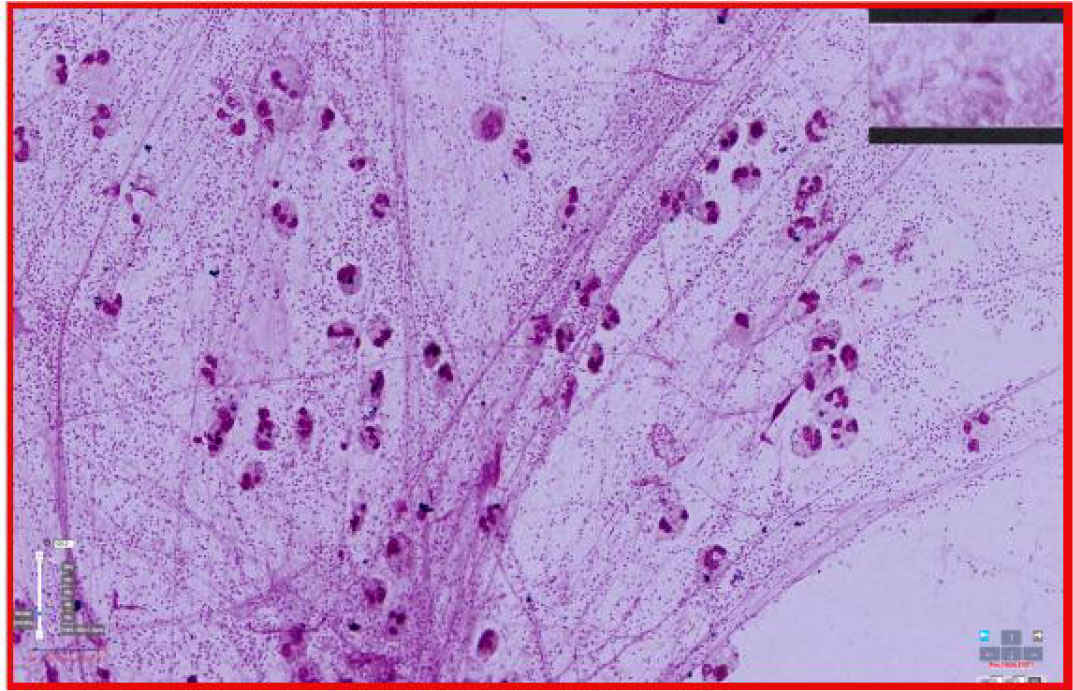
(Right) Qualifying sputum smear, with no visible significant squamous epithelial cells (×3700 magnification).

a. Sputum sample adequacy was assessed by examining the numbers of polymorphonuclear leukocytes and squamous epithelial cells in bedside M-ROSE smears. The criteria are as follows:

- A qualified deep lower respiratory tract sputum sample was defined as having more than 25 polymorphonuclear leukocytes and fewer than 10 squamous epithelial cells per low-power field (LPF), or a squamous epithelial cell–to–leukocyte ratio of less than 10%.
- Samples containing more than 10 squamous epithelial cells, or a squamous epithelial cell ratio exceeding 10%, were considered contaminated with saliva or upper respiratory secretions and were excluded from further analysis (Chinese Thoracic Society Respiratory and Critical Care Medicine Group and Chinese Medical Doctor Association Respiratory Critical Care Medicine Committee, 2020).Examples are shown in **Figures 1** and **2**:

b. Stained sputum M-ROSE smears were scanned using the Bionovation 800MP digital scanner at 100× magnification, corresponding to approximately 3700× under light microscopy. A total of 161 images containing Acinetobacter baumannii, Klebsiella pneumoniae, and Pseudomonas aeruginosa were acquired, with each image measuring 2448 × 2048 pixels. The bacterial species present in all smears were confirmed by microbial mass spectrometry.

##### 1.1.1.2 Annotation

Using Labelme software, an arbitrary-point annotation method was employed to annotate individual bacteria in 30 selected sputum M-ROSE smear images.

## 2 Methods

**Figure 3:**
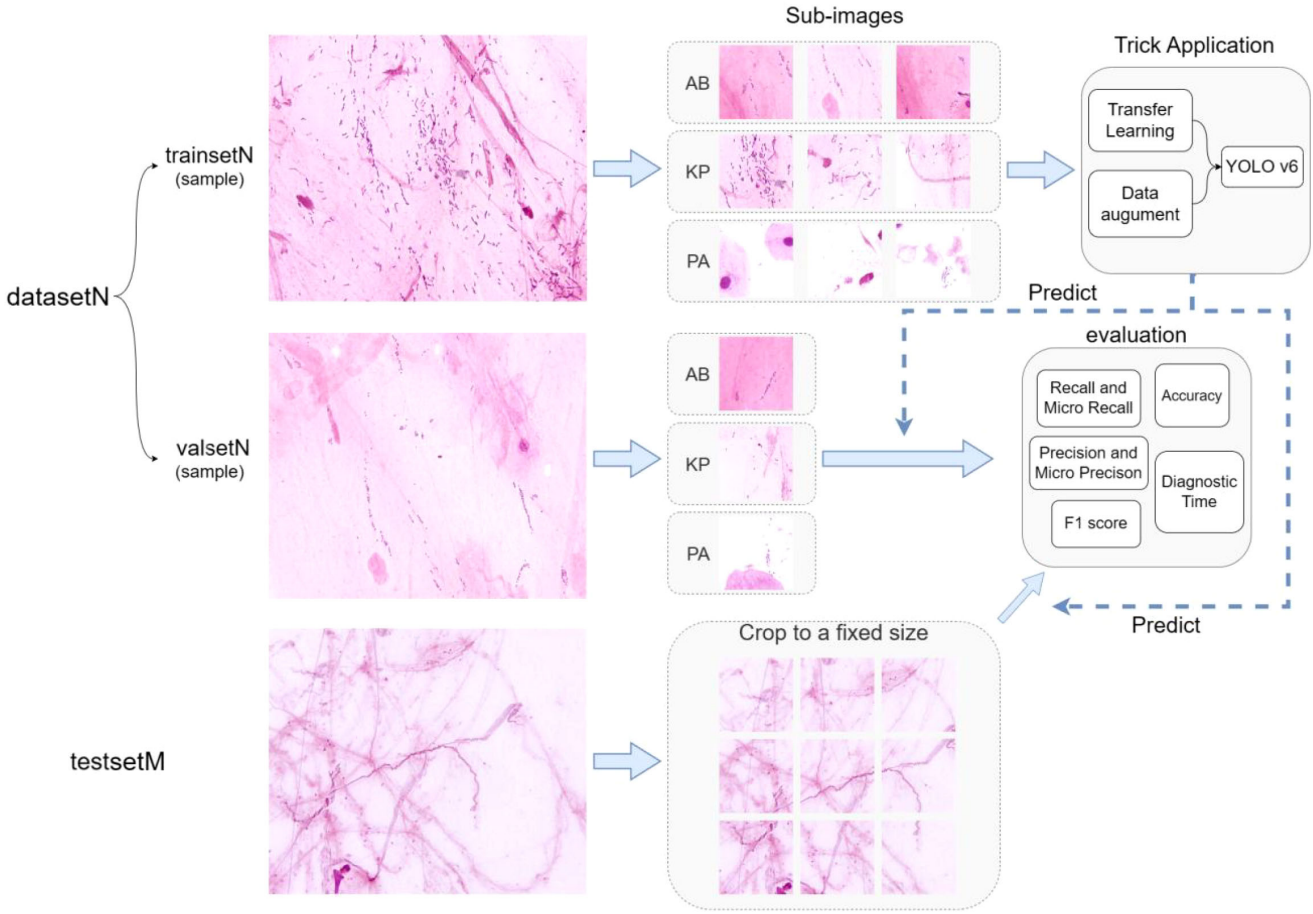
Overview of the methods.

The data processing and overall operational workflow for the different datasets used in this study are shown in **Figure 3**.

### 2.1 Data setup and partitioning

A total of 161 sputum M-ROSE smear images scanned at 100× magnification were collected for this study. From these, 10 images each containing Acinetobacter baumannii (AB), Klebsiella pneumoniae (KP), and Pseudomonas aeruginosa (PA) were selected to construct Dataset N. Bacterial targets in these images were individually annotated by experienced physicians using the arbitrary-point annotation method. Dataset N was then randomly divided into training and validation sets at a ratio of 8:2. These subsets were used to train and validate the deep learning model for bacterial localization, identification, and classification.

**Figure 4:**
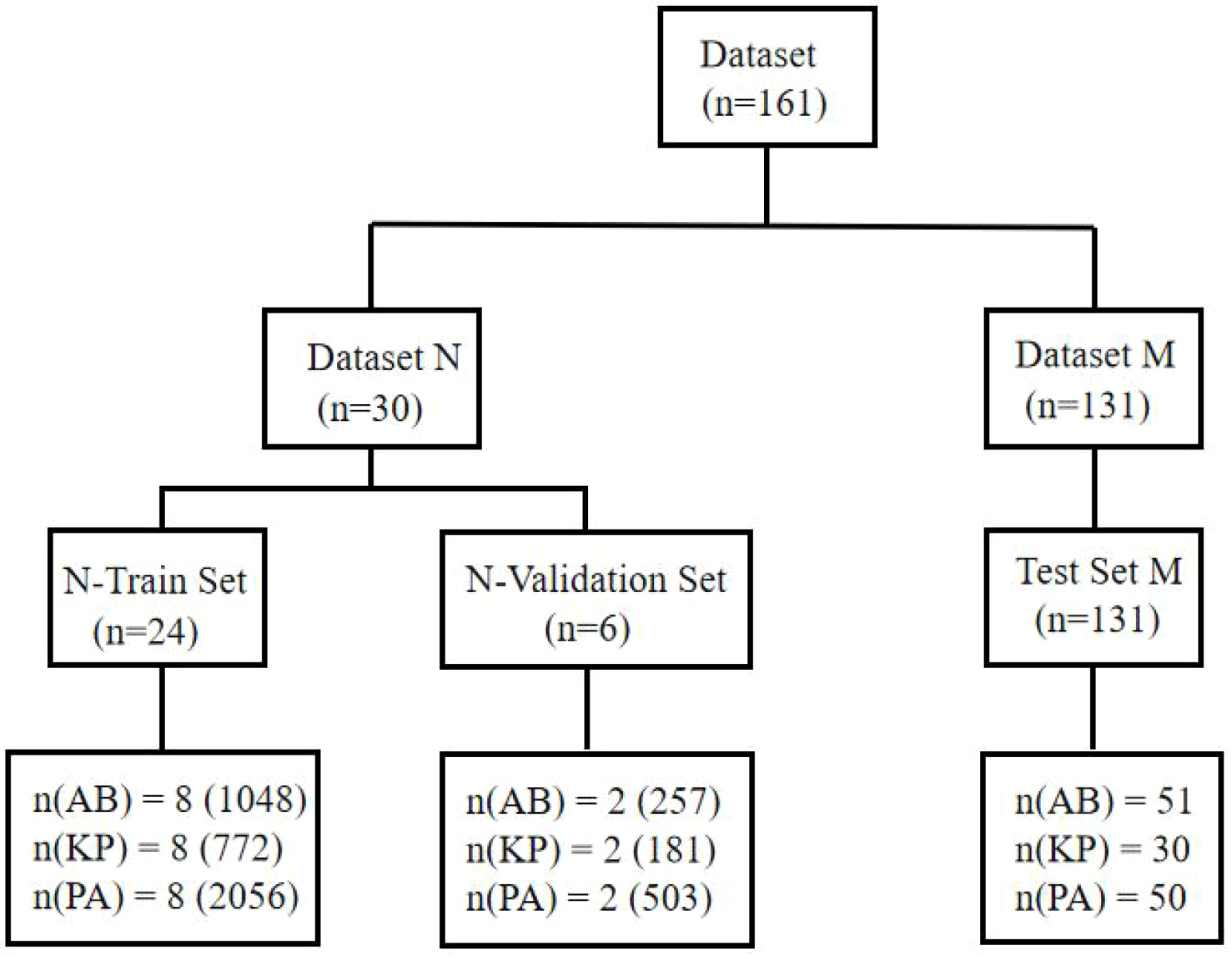
Partitioning of the dataset.

The remaining 131 images were designated as Test Set M and were used to evaluate the model’s ability to identify bacterial species in sputum smears. The dataset partitioning is shown in **Figure 4**.

In the sputum M-ROSE smear data, the size of the bacteria typically measures around 10 × 10 pixels, accounting for approximately 0.002% of the original image, making detection extremely challenging. The bacteria in sputum M-ROSE smears often exhibit linear or clustered distributions, with occasional adhesion, resulting in highly irregular distribution patterns. This further complicates the tasks of locating and identifying the bacteria.

**Figure 5:**
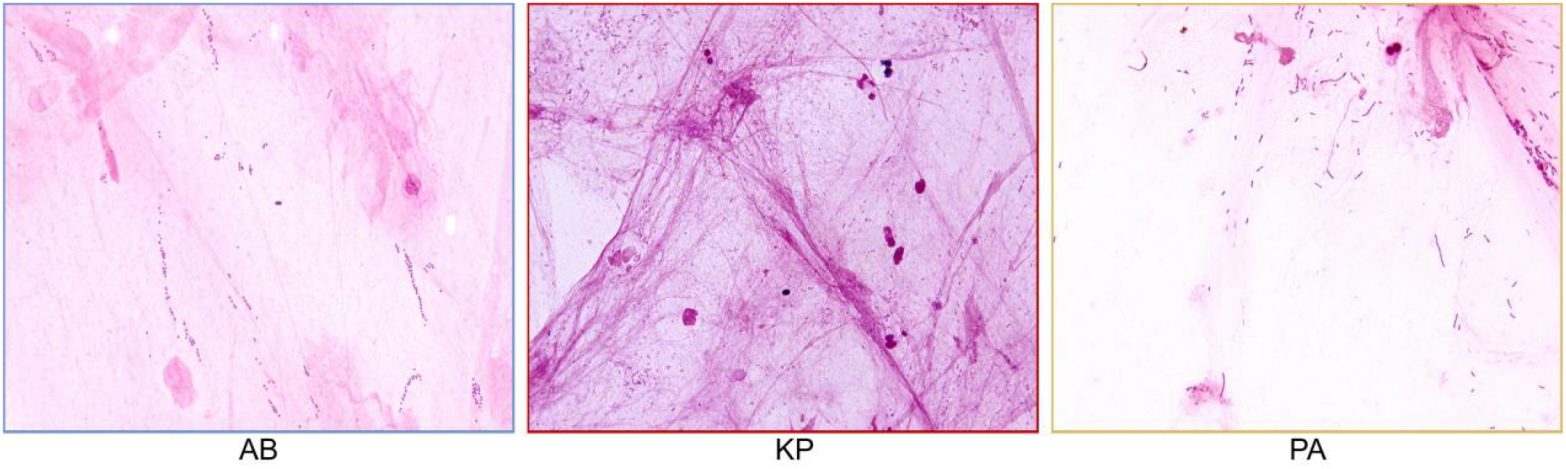
Sputum M-ROSE Smears (×3700 magnification).

The 100× magnified images of sputum M-ROSE smears infected with the three types of bacteria are shown in **Figure 5**.

### 2.2 Transfer learning

Existing deep learning methods generally depend on large amounts of training data. Generally, the scale of the model determines its ability to handle tasks of varying complexities because the model’s expressive capacity must be sufficiently large to uncover underlying patterns in the data (Tan et al., 2018). Moreover, the relationship between model size and the amount of data required for training is largely linear (Ribani and Marengoni, 2019). However, in the medical field, where data annotation costs are high, it is often impractical to train large-scale deep neural networks from scratch due to insufficient data.

Because no large-scale public dataset is currently available for bacterial annotation in sputum M-ROSE smears, fine-tuning–based transfer learning was adopted in this study. A YOLOv6 object detection model pretrained on the COCO dataset, which contains approximately 160,000 labeled images, was adapted for bacterial detection in sputum M-ROSE smears. Because bacteria in these smears are small targets, the YOLOv6s variant, which is optimized for small-object detection, was selected.

**Figure 6:**
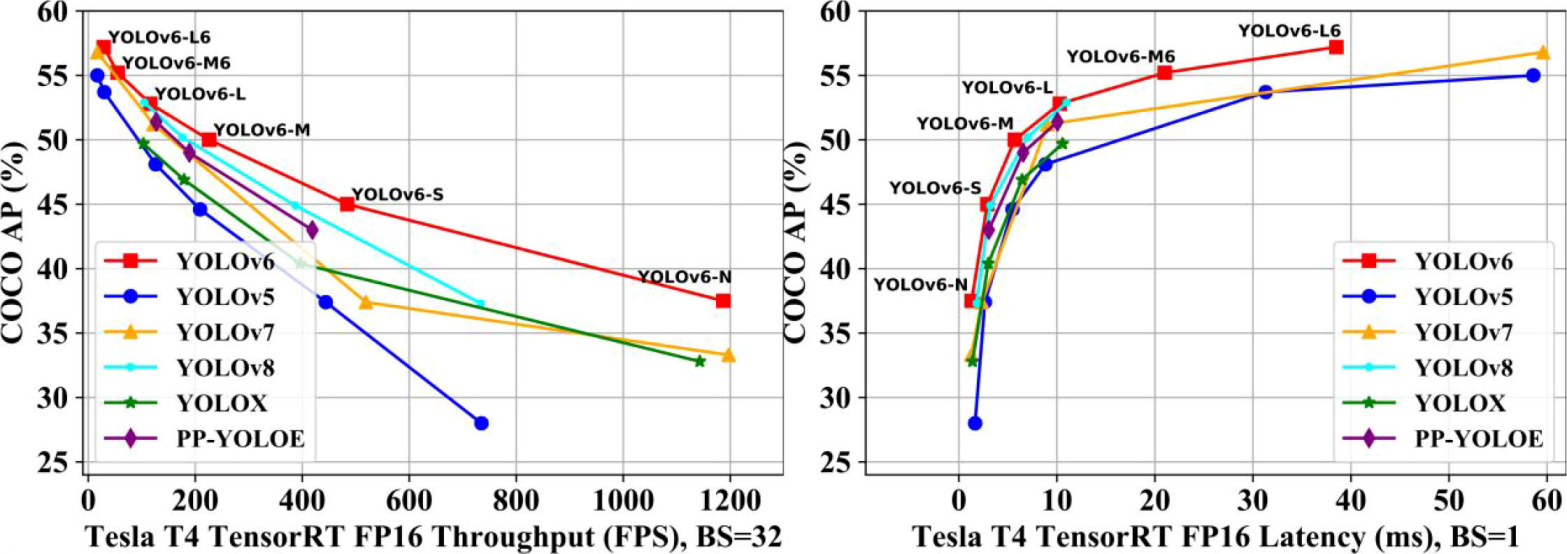
Performance comparison of mainstream efficient object detection models (Li et al., 2022).

The YOLO (You Only Look Once) series has become one of the most popular detection frameworks in industrial applications due to its excellent balance between speed and accuracy (Girshick et al., 2014; Fang et al., 2018; Wu et al., 2019; Tamiev et al., 2020; Zhao et al., 2020; Wang and Bu, 2021; Fu et al., 2022; Zhang et al., 2022). YOLOv6 can rapidly and accurately detect multiple objects in images or videos, and the optimized version of YOLOv6 surpasses all known object detectors in both speed and accuracy, achieving performance between 10 FPS and 160 FPS. It also boasts the highest accuracy among all known real-time object detectors on the COCO dataset at 10 FPS or higher, with an average precision (AP) of 57.2% (Li et al., 2022; Wang et al., 2022), as shown in **Figure 6**.

### 2.3 Data augmentation in few-shot learning

Considering the characteristics of the original data, this study combined data augmentation techniques with transfer learning and few-shot learning to meet the data requirements of the deep learning model. After evaluating the characteristics of sputum M-ROSE smears and the intended application context, we implemented several conventional augmentation methods, including image flipping, right-angle rotation, noise addition, Gaussian blurring, and adjustments in brightness, contrast, and saturation.

In addition, we proposed specialized augmentation techniques tailored to the unique attributes of sputum M-ROSE smear data:

#### 2.3.1 Rotation cutting method

Conventional cropping methods effectively address the issue of large image sizes, but they generate relatively few images, resulting in limited data augmentation effects. Standard rotation methods involve rotating the image and then filling in the empty areas to restore it to a square shape. The rotated image ends up larger than the original, with four triangular voids around the edges. Even if resized or center-cropped back to the original dimensions, some information is lost, which is detrimental to model training.

We further introduced a novel cropping strategy suitable for direction-invariant bacterial images, referred to as the rotation cutting method. Unlike conventional sliding-window cropping, this technique exploits the direction invariance of bacterial images by incorporating rotation angle as a third variable in addition to the horizontal and vertical strides. This approach not only addresses the problem of large image size but also enhances the effectiveness of conventional cropping-based augmentation.

**Figure 7:**
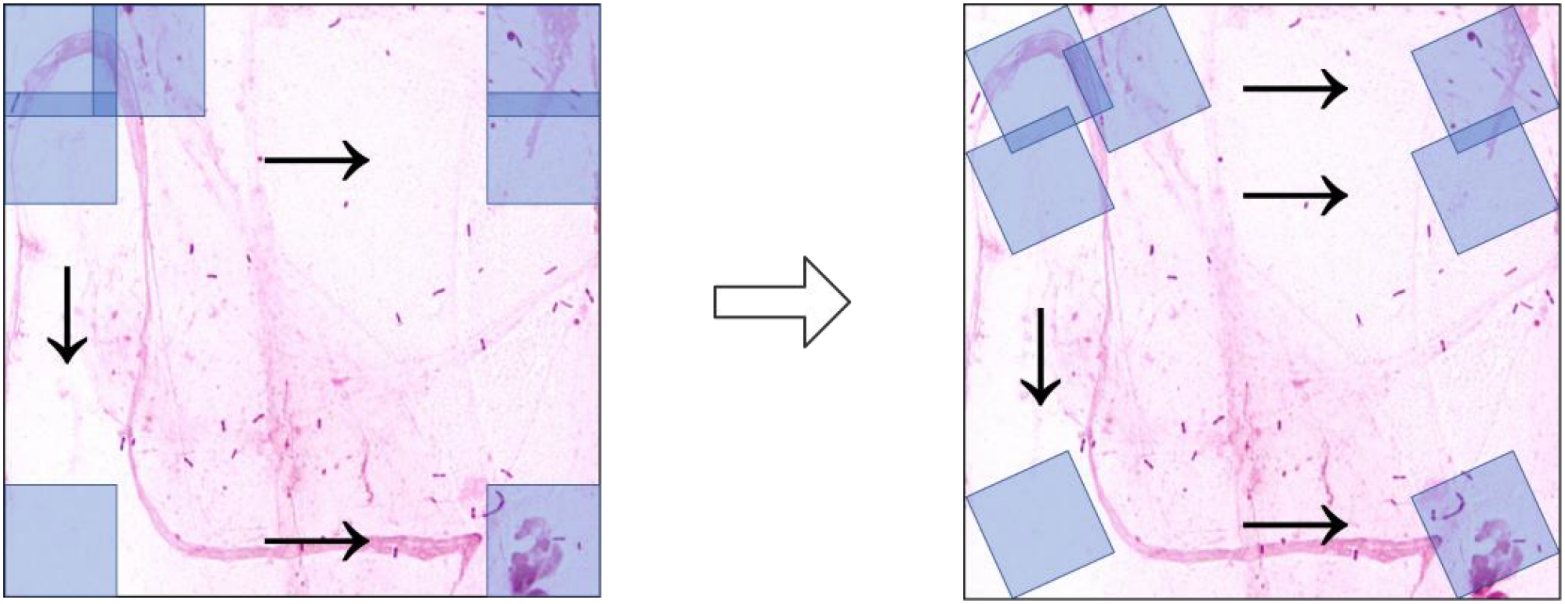
Schematic comparison of the conventional cutting method and the rotation cutting method.

As shown in **Figure 7**, this innovative rotation cutting method significantly increases the diversity of the dataset while maintaining the integrity of the original information.

In implementing arbitrary angle rotation and restoration mapping of the cutting box, we also achieved synchronous rotation mapping of the annotated data coordinates through mathematical methods, completing the encapsulation of the rotation cutting method.

**Figure 8:**
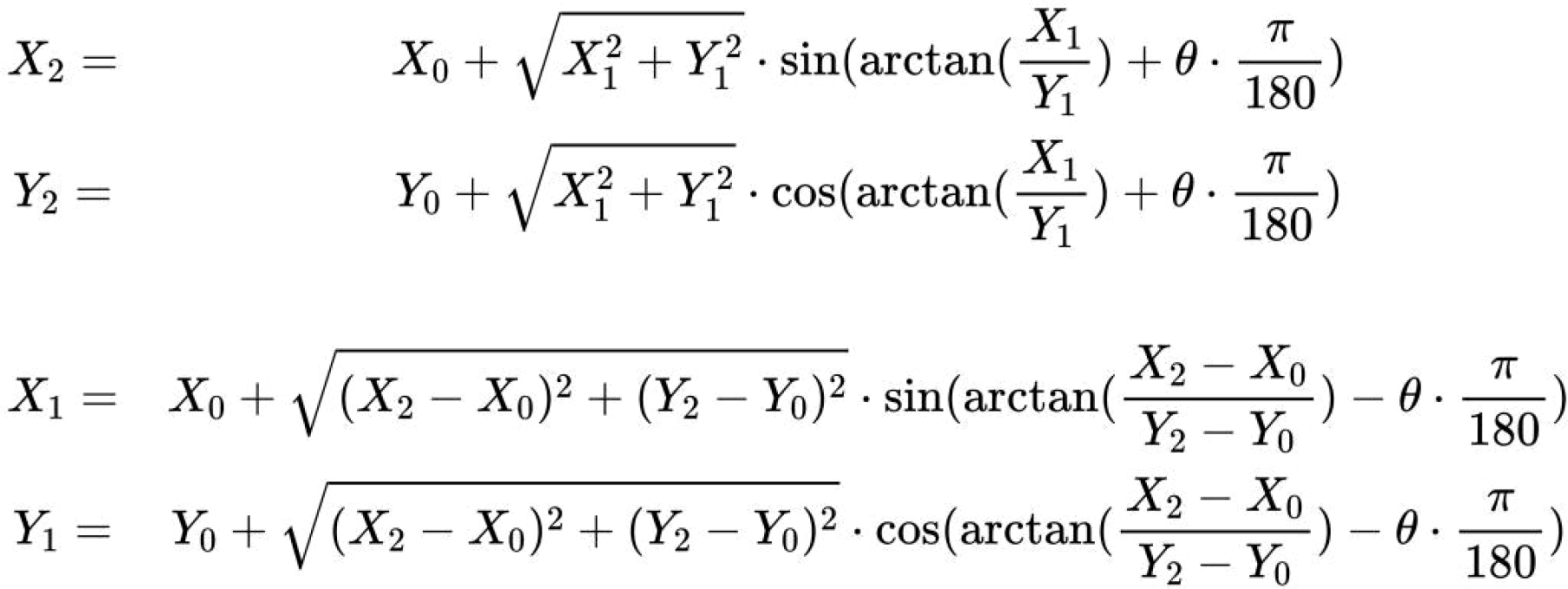
Mapping formulas for annotated coordinates after rotation cutting.

Considering the top left vertex of the image as the origin, with width as the Y-axis and height as the X-axis, let the coordinates of the top left vertex of the small image in the original image be (X0, Y0), the coordinates of the annotation point in the small image be (X1, Y1), and the coordinates in the original image be (X2, Y2). Given the rotation angle θ, the mutual mapping formulas between (X2, Y2) and (X1, Y1) are as follows (illustrated in **Figure 8**).

#### 2.3.2 Image fusion method

**Figure 9:**
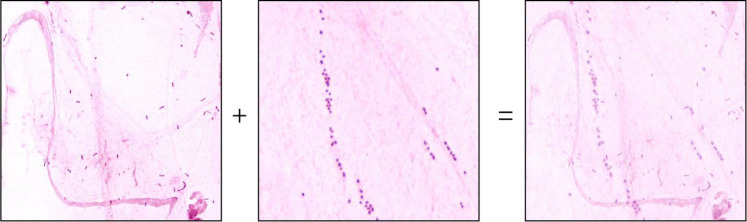
Schematic diagram of image fusion.

Image fusion was used as a specialized augmentation technique in which multiple images were combined to create new training samples. By taking the minimum RGB-channel values across multiple images, bacterial patterns from different images could be merged into a single synthetic image, as illustrated in **Figure 9**.

The advantages of this method include:

##### 2.3.2.1 Improved classification focus

The original images primarily contained only one bacterial type. If the model were trained exclusively on these images, it might tend to classify whole-image patterns rather than individual bacterial targets. However, training with the fused images, which include multiple types of bacteria, encourages the model to focus on classifying by the bacterial units, enhancing its classification capabilities.

##### 2.3.2.2 Reduced environmental bias

Given the limited data, the environment surrounding the bacteria in the images tends to be quite fixed. During training, the model may inadvertently link recognition results to the overall environment of the images. Image fusion was intended to reduce this environmental bias, encouraging the model to focus more on bacterial morphology itself rather than on surrounding background features.

##### 2.3.2.3 Simulated adhesion scenarios

The original data features few complex cases of bacterial adhesion, making it difficult for the model to learn how to differentiate between adhering bacteria effectively. By using image fusion, we artificially generated numerous adhesion-like scenarios. While these are not as natural as actual adhesions, they still enhance the deep learning model’s ability to handle such complexities.

Because most clinical smears primarily involve infection by a single bacterial type, the generation parameters for fused images were set to 80% same-type bacteria and 20% different-type bacteria.

## 3 Experiments and results

### 3.1 Training environment and parameter settings

This study was implemented using a Linux operating system, the PyTorch deep learning framework, and Python 3.9. The experiments were conducted on a workstation equipped with an Intel Xeon E5–2650 v4 CPU and an NVIDIA GeForce GTX 1080 Ti GPU. During training, transfer learning was applied using YOLOv6s as the pretrained model. The Adam optimizer was used, with a batch size of 16, an initial learning rate of 0.0003, and a total of 40 epochs. Model weights were saved after each epoch, and the model with the best performance was selected for final evaluation.

### 3.2 Evaluation metrics

Model performance was assessed from two perspectives: detection performance and clinical effectiveness. Detection performance was evaluated using Recall (R), macro-Recall (macro-R), Precision (P), macro-Precision (macro-P), F1 score, and mean Average Precision (mAP). According to previous criteria, bacteria observed at >20 organisms per oil-immersion field or accounting for >50% of all bacteria in a smear may represent the predominant pathogen (Garcia, 2010; Huang et al., 2020). Accordingly, in this study, an image was classified as infection by a given bacterial species when that species accounted for more than 50% of all detected bacteria in the image. Predictions consistent with the reference standard were recorded as T, whereas inconsistent predictions were recorded as F. Clinical applicability was assessed using accuracy (ACC) and diagnostic time (DT), with comparison against physician interpretation.

### 3.3 Experimental setup

To evaluate the effectiveness of rotation cutting and image fusion, three training datasets—A, B, and C—were generated from the training subset of Dataset N, each containing 50,000 images. Dataset A used standard data augmentation, Dataset B included rotation cutting, and Dataset C incorporated image fusion.

**Figure 10:**
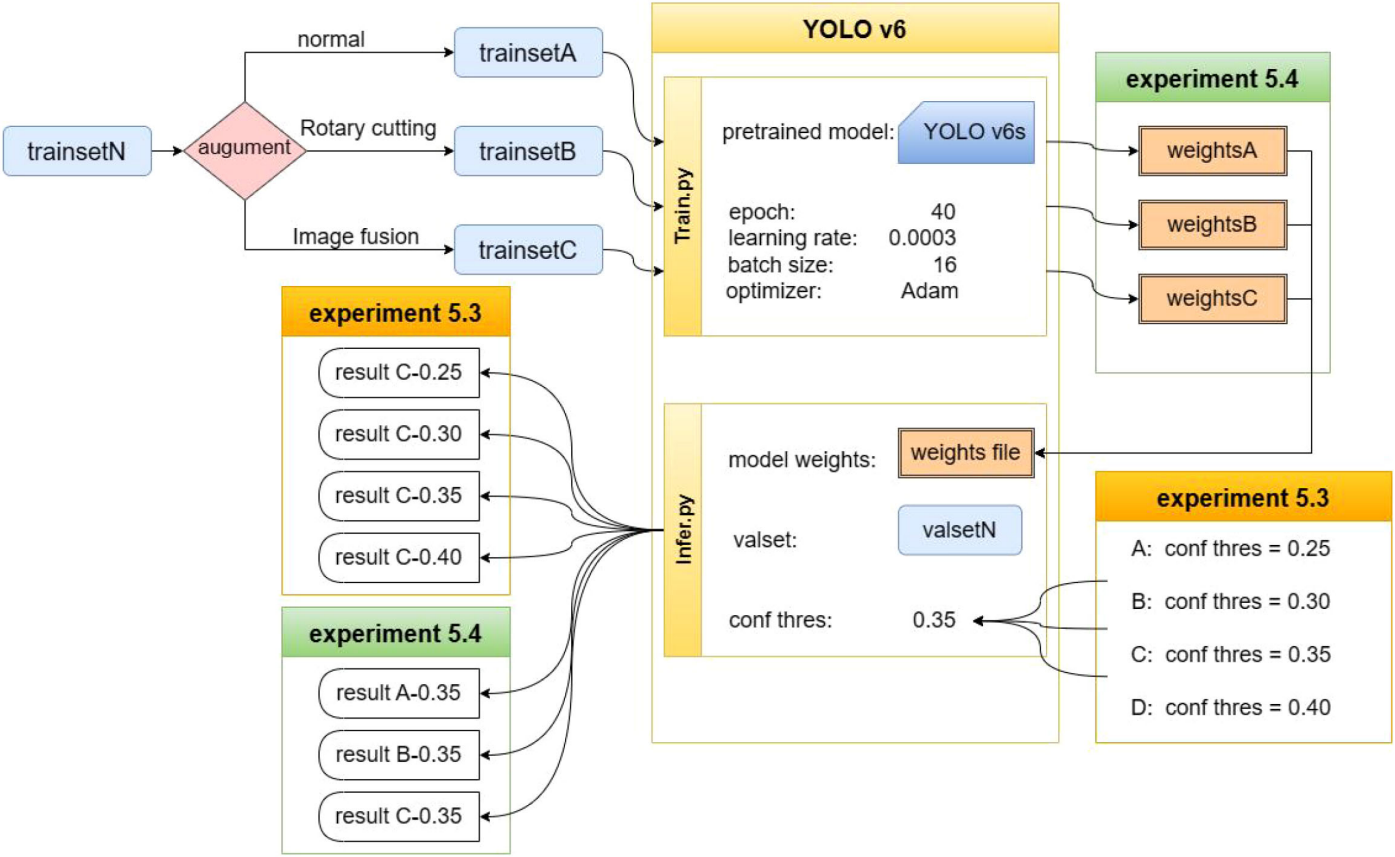
Experimental setup and training workflow.

The model trained on Dataset C was then evaluated on the validation set using confidence thresholds of 0.25, 0.30, 0.35, and 0.40 to compare detection performance across thresholds. Finally, the model trained on Dataset C was tested on Test Set M at a confidence threshold of 0.35, which yielded the best detection performance, and its accuracy and efficiency were compared with manual diagnosis. The specific experimental setup and training workflow are illustrated in **Figure 10**.

### 3.4 Results

To evaluate the effectiveness of the two specialized augmentation methods—rotation cutting and image fusion—we compared model performance in terms of localization and classification before and after their application, as shown in **Table 1**. In this comparison, the confidence threshold was set at 0.35, which had previously been shown to yield the best performance.

**Table 1:** Comparison of data augmentation effects.

Data augmentation	R	macro-R	P	macro-P	F1	mAP
None	R_0_ = 11.15%	38.08%	P_0_ = 100.0%	92.64%	0.5397	0.252
	R_1_ = 14.29%		P_1_ = 100.0%			
	R_2_ = 88.81%		P_2_ = 77.91%			
+Normal	R_0_ = 93.93%	84.51%	P_0_ = 81.91%	82.85%	0.8367	0.783
	R_1_ = 80.57%		P_1_ = 82.94%			
	R_2_ = 79.02%		P_2_ = 83.70%			
+Rotary cutting	R_0_ = 95.30%	88.02%	P_0_ = 79.84%	82.74%	0.8530	0.791
	R_1_ = 77.14%		P_1_ = 83.85%			
	R_2_ = 91.61%		P_2_ = 84.52%			
+Image fusion	R_0_ = 95.11%	89.31%	P_0_ = 83.22%	84.23%	**0.8670**	**0.817**
	R_1_ = 77.71%		P_1_ = 85.00%			
	R_2_ = 95.10%		P_2_ = 84.47%			

Bold values indicate the best performance among the compared methods.

Additionally, in this study, we asked Doctor A (with 3 years of clinical experience) and Doctor B (with 5 years of clinical experience) to independently diagnose the 131 images in the test set M without prior knowledge of the smear results. Their diagnoses were then compared with the standard results to calculate the accuracy (ACC). As mentioned earlier, considering that humans cannot maintain focused attention for extended periods, the calculation of manual diagnosis time in this study was conducted in batches, with the total time for each batch being summed. Each batch consisted of 10 images. For comparison with the model’s decision time, we also recorded the average time taken by both doctors to diagnose a batch of 10 images. The comparison results are shown in **Table 2**.

**Table 2:** Comparison of diagnostic efficiency between neural network model and clinicians.

	Accuracy (%)	Diagnostic time for 131 images (s)	Diagnostic time per 10 images (s)
Doctor A	88.55%	8253	635
Doctor B	92.37%	6681	510
YOLOv6-s	**93.89%**	**231**	**109**

Bold values indicate the best performance among the compared methods.

## 4 Discussion

In recent years, deep learning has been increasingly applied in medicine. The integration of artificial intelligence with multiple disciplines has stimulated methodological innovation and expanded its potential applications in medicine (Kim et al., 2022; Muneeb et al., 2022; Nguyen et al., 2022; Pereira and Silveira, 2022). This study employed a deep learning approach integrating transfer learning and few-shot learning. YOLOv6 served as the base framework, and YOLOv6s was used as the pretrained model for bacterial detection in sputum M-ROSE smears. The model achieved an F1 score of 0.8670, indicating good performance in detecting small bacterial targets. Comparison with two physicians showed that the proposed model achieved bacterial identification accuracy comparable to that of Physician B and higher than that of Physician A, supporting its potential clinical utility.

### 4.1 Analysis of results under different data augmentation methods

Without data augmentation, the model showed poor detection performance, with a macro-recall of only 38.08%, indicating that the original dataset was insufficient for stable fine-tuning under transfer learning. After standard data augmentation was applied, macro-recall improved to 84.51% and mAP increased to 0.783, suggesting that the augmented data were sufficient to support model fine-tuning. When rotation cutting was added, mAP improved slightly, but the overall gain was modest. Two factors may explain this finding. First, standard augmentation already included right-angle rotation; therefore, although rotation cutting generated more image patches with finer directional variation, part of the additional directional information may have overlapped with that provided by standard augmentation. Second, although rotation cutting was expected to preserve more information than conventional rotation-based cropping, the gain in informative edge content appeared to be limited. Taken together, these factors may explain why rotation cutting did not yield the expected improvement. By contrast, image fusion increased mAP by 2.6 percentage points, indicating a meaningful performance improvement. This finding suggests that image fusion effectively reduced environmental bias and encouraged the model to focus more on bacterial morphology. Ultimately, by combining transfer learning with multiple augmentation strategies, the YOLOv6 model achieved an mAP of 0.817. This level of performance indicates that the model can support accurate bacterial classification in sputum M-ROSE smears through fine-grained object detection.

### 4.2 Performance analysis of the neural network model compared to clinical physicians

The trained YOLOv6 model achieved 93.89% diagnostic accuracy on Test Set M, which was comparable to that of Physician B and higher than that of Physician A. This finding indicates that the YOLOv6 model can identify bacteria in sputum smears with good accuracy and may provide useful support for clinical interpretation. In addition, the model required only 231 seconds to analyze 131 images, whereas the two physicians required approximately 2 hours in total, highlighting its substantial efficiency advantage. Computer-assisted image assessment may reduce clinician workload and facilitate more timely clinical decision-making.

Additionally, this study recorded the time required for each step of computational diagnosis. When all 131 images were analyzed simultaneously, data preprocessing required 31 seconds, model loading and batch normalization–convolution fusion required 92 seconds, inference required 87 seconds, and result conversion and saving required 21 seconds, for a total of 231 seconds. When 10 images were analyzed, preprocessing required 6 seconds, model loading and fusion required 92 seconds, inference required 8 seconds, and result conversion and saving required 3 seconds, for a total of 109 seconds. These findings suggest that the computational advantage becomes more evident when larger numbers of images are processed, and that the average detection time for small image batches cannot be estimated by simple division. In future work, the framework could be extended to include additional bacterial species and further developed into a rapid identification platform that may also support remote clinical guidance.

## Data Availability

The original contributions presented in the study are included in the article/**Supplementary Material**. Further inquiries can be directed to the corresponding author.
