# Supplementary material for: Application of few-shot learning and transfer learning based on YOLOv6 in the recognition of bacteria in sputum M-ROSE
Source: Front Cell Infect Microbiol. 2026 Apr 22;16:1791986. doi: 10.3389/fcimb.2026.1791986 (PMC13143893; doi:10.3389/fcimb.2026.1791986)
Supplement: Supplementary file 1 [file DataSheet1.docx]

Attachment 1: Standardized Procedure for Laboratory Preparation of Sputum M-ROSE Specimens

The collection, preparation of smears, staining, and microscopy of sputum M-ROSE specimens are carried out according to a standardized procedure established by the laboratory.

## 1.1 Collection and Handling of Sputum Specimens

**1.1.1 Collection and Storage of Sputum Specimens:**
Sputum specimens are collected using a sputum collector with negative pressure suction, obtaining two samples of deep lower respiratory tract sputum from patients in the ICU with tracheal intubation or tracheostomy. After collection, one sample is placed in a refrigerator for cold storage and processed within one hour, followed by subsequent smearing and testing. The other sample is immediately sent to the laboratory for culture and microbial mass spectrometry identification.

**1.1.2 Preparation of Sputum M-ROSE Smears:**
The preparation of sputum M-ROSE smears is performed inside a biosafety cabinet. On a sterile workbench, take a clean, dry glass slide and wipe its surface with a sterile cotton ball or swab to ensure it is free from grease and impurities.

**1.1.3 Taking a Small Amount of Sputum:**
Using a sterile cotton swab, take a small amount of sputum from the sample and evenly spread it on the left side of the glass slide in an oval shape. Ensure that saliva or other contaminants from the mouth do not mix with the sputum. Select three representative small pieces of sputum from different areas of the specimen for slide preparation.

**1.1.4 Smearing the Sputum:**
Hold the slide with sputum slightly heated in front of a flame or sterilizing infrared lamp with the left hand, while the right hand takes another cold slide and places it in a "X" shape to trap the sputum between the two slides. Press to spread it fully, ensuring not to release the pressure, and slowly drag to the right at a constant speed to complete the sputum slide preparation, avoiding smears that are too thick or too thin.

**1.1.5 Drying:**
Allow the smear to air dry at room temperature for about 10 minutes, or heat it back and forth over an alcohol lamp flame to promote sputum drying. Ensure the smear is completely dry for adequate penetration of the stain.

**1.1.6 Fixation:**
Fix the smear using the flame of an alcohol lamp. Heat the slide several times over the flame to ensure bacteria adhere to the slide and are fixed.

## 1.2 Gram Staining and Microscopy of Sputum M-ROSE Smears

**1.2.1 Staining:**
a. Apply 0.5ml-0.8ml of crystal violet stain to the smear, using an ear syringe to blow a gentle breeze to cover the entire surface of the slide. After 10 seconds, rinse with running water for 1-2 seconds and blot with paper;
b. Apply 0.5ml-0.8ml of iodine solution to the smear, again using an ear syringe to ensure complete coverage. After 10 seconds, rinse with running water for 1-2 seconds and blot with paper;
c. Use acetone-alcohol decolorizer to decolorize the smear until it is colorless, then rinse with running water for 1-2 seconds and blot with paper;
d. Stain with safranin solution for 10-20 seconds, rinse with water for 1-2 seconds, and blot with paper. Allow to dry before microscopy. (During rinsing, do not pour out the dye; it should be rinsed with running water to prevent sediment from settling on the specimen.)

**1.2.2 Drying:**
Place the stained smear at room temperature or use a warming cabinet to ensure it is completely dry for observation under a microscope.

**1.2.3 Microscopy:**
Assess the quality of the sputum M-ROSE smear. Qualified lower respiratory tract deep sputum M-ROSE smears will proceed to subsequent scanning and reading.
